# Role of paracrine factors in stem and progenitor cell mediated cardiac repair and tissue fibrosis

**DOI:** 10.1186/1755-1536-1-4

**Published:** 2008-10-13

**Authors:** Jana S Burchfield, Stefanie Dimmeler

**Affiliations:** 1 Institute of Cardiac Regeneration, Center of Molecular Medicine, University Frankfurt, Theodor Stern Kai, 60590 Frankfurt, Germany

## Abstract

A new era has begun in the treatment of ischemic disease and heart failure. With the discovery that stem cells from diverse organs and tissues, including bone marrow, adipose tissue, umbilical cord blood, and vessel wall, have the potential to improve cardiac function beyond that of conventional pharmacological therapy comes a new field of research aiming at understanding the precise mechanisms of stem cell-mediated cardiac repair. Not only will it be important to determine the most efficacious cell population for cardiac repair, but also whether overlapping, common mechanisms exist. Increasing evidence suggests that one mechanism of action by which cells provide tissue protection and repair may involve paracrine factors, including cytokines and growth factors, released from transplanted stem cells into the surrounding tissue. These paracrine factors have the potential to directly modify the healing process in the heart, including neovascularization, cardiac myocyte apoptosis, inflammation, fibrosis, contractility, bioenergetics, and endogenous repair.

## Heart failure and stem cells

Although coronary artery disease accounts for two-thirds of heart failure cases in the United States [1], other causes leading to heart failure are due to non-ischemic events and include myocarditis, hypertension, diabetes, arrhythmias, valvular disease, hypothyroidism, and drug-induced cardiotoxicity. The molecular and cellular mechanisms mediating heart failure have been the focus of numerous research efforts, and include cardiac myocyte apoptosis and necrosis, cardiac myocyte hypertrophy, interstitial fibrosis, decreased contractility, inflammation, oxidative stress, and impaired neovascularization. Pharmacological therapies for the treatment of heart failure have traditionally targeted pump function and quality of life for end-stage heart failure patients, and although several medications are available to limit the progression of the disease, the current therapies or interventional procedures do not lead to replacement of tissue and, thus, do not stop or reverse progression of adverse left ventricular (LV) remodeling in all patients [2,3]. The use of stem cell-based therapy is becoming increasingly recognized as having the potential to salvage damaged myocardium and to promote endogenous repair of cardiac tissue [4-6]. Although the available data in this area are highly debatable, the potential of stem cell-based therapy for the treatment of heart failure remains an alternative option.

Stem cells are defined as cells that have the capacity to self renew, multipotency/pluriopotency, and clonality, and are divided into embryonic stem cells and adult stem cells. Although embryonic stem cells may have more potential for cardiac differentiation and thus replacement of damaged myocardium, few studies have focused on paracrine factors released from these cells that may be involved in mediating cardiac repair. Therefore, this review will focus on adult stem or adult progenitor cells, since numerous studies suggest that paracrine factors released from these cells may comprise an important mechanism contributing to cardiac protection after their transplantation into the myocardium.

## Types of stem cells

Adult stem cells comprise at least three different groups: bone marrow-derived stem cells, the circulating pool of stem or progenitor cells, which, at least in part, are derived from the bone marrow, and tissue-resident stem cells. Bone marrow contains a complex assortment of progenitor cells, including hematopoietic stem cells (HSCs), so-called 'side population cells' (SP cells; defined by the expression of the Abcg2 transporter, which enables export of a Hoechst dye) [7], mesenchymal stem cells (MSCs) or stromal cells [8], and multipotential adult progenitor cells (MAPCs), a subset of MSCs [9], (see Table 1).

**Table 1:** Summary of cell derived factors in different cell populations.

Stem cell type	Stem-cell derived factors
**Bone marrow**	
BM-MNCs	VEGF, bFGF, Ang-1, IL-1β, TNF-α [26]
BMSCs (HSCs and MSCs)	VEGF, bFGF, IGF, SDF-1, Akt, eNOS [27]
MSCs	VEGF, bFGF, Ang-1, IL-1, SDF-1, PIGF, MCP-1, FGF-7, IL-6, TGF-β, PDGF, PA, MMP-9, TB4, Sfrp, Tenacin C, Thrombospondin-1 [28,29]
MAPCs	VEGF, MCP-1, TGF- β, PDGF-BB [32]
Multipotent BM-derived cells (non-HSCs and non-MSCs)	VEGF, bFGF, Ang-1, Ang-2, IGF-1, SDF-1α, PDGF-B, HGF [34]
	
**Circulating**	
Cultured PB-MNCs	VEGF, HGF, G-CSF, GM-CSF [35]
EPCs	VEGF-A, VEGF-B, SDF-1, IGF-1 [72]; VEGF-A FGF-2, IGF-1, HGF, Ang-1, SDF-1 [36]
	
**Tissue resident**	
Cardiac stem cells	
c-kit^+^, MDR-1^+^, Sca-1^+^	IGF-1, HGF [71]
Adipose stem cells	MMP-9, MMP-3 [82]; VEGF, HGF, TGF-β [83,84]

Another population of progenitor cells that has also been shown to have therapeutic potential is the pool of progenitor cells circulating within the blood. Asahara and Isner isolated the so called 'endothelial progenitor cells' (EPCs), defined by their function to form new blood vessels and enhance neovascularization after ischemia (for a review, see [10,11]. However, the definition of these cells has recently been challenged [12] and it appears that these cells, isolated and cultured from the mononuclear cell fraction, may actually consist of a mixture of cells including HSCs, EPCs, and myeloid cells. Regardless of the exact definition of these cell populations, it appears that these cells have the capacity to acquire an endothelial-like phenotype, or at least, have the capacity to promote neovascularization.

Tissue-resident stem cells in the heart, the 'cardiac stem' cells, include cells characterized by c-Kit^+ ^marker expression [13], Sca-1^+ ^marker expression [14], cardiac SP cells [15] and cells expressing the protein Islet-1 [16]. Another type of cardiac stem cell has been identified by growing self-adherent clusters (termed 'cardiospheres') from subcultures of murine or human biopsy specimens. Whether these cells and c-Kit^+^, Sca-1^+ ^and cardiac SP cells comprise distinct cell populations is not entirely clear. Furthermore, the exact origin of these c-Kit^+^, Sca-1^+^, SP, Islet-1^+^, or cardiosphere-derived cardiac stem cells and the mechanisms maintaining the cardiac stem cell pool are unclear. Two recent studies suggest that c-Kit^+ ^and cardiac SP cells may arise from the bone marrow [17,18]. However, these studies cannot entirely exclude that specific subpopulations of cardiac stem cells originate from the heart and these cardiac stem cells may represent remnants from embryonic development in selected niches within the heart.

## Clinical trials

Most of the clinical trials using stem/progenitor cells to treat patients following an ischemic event have, to date, used cells derived from the bone marrow [19-22]. Using a systematic review and meta-analysis of all of the completed clinical trials using bone marrow-derived stem cells to treat ischemic heart disease, Abdel-Latif *et al*. [23] and Lipinski *et al*. [24] suggested that the transplantation of these cells is safe and efficacious beyond the benefits achieved using traditional therapy using pharmaceuticals. Using such analysis, these studies found that there were decreases in infarct scar size, improvements in ejection fraction, and decreased left ventricular end systolic volume, suggesting improvement in overall global function. Interestingly, these studies found no significant differences in outcomes with the use of less or more than the median number of transplanted stem cells; however, since most of the clinical trials used different cell isolation protocols and subsets of bone marrow-derived cells, it remains unclear which cell subpopulations would have the most beneficial effects [25].

While it is virtually impossible to define the precise mechanisms involved in bone marrow cell-mediated improvement in LV function in patients, the use of animal models of heart disease aids not only in the discovery of which stem cell population is the most efficacious, but also in determining whether there are overlapping or differential mechanisms between stem cell populations, such as the release of paracrine factors. Paracrine factors, such as growth factors and cytokines, are normally released from endogenous cells of the heart in response to injury. These factors may signal via the circulation to mediate stem cell homing from the circulation, bone marrow, or tissue to the site of injury, thus aiding in tissue repair. As a focus of this review, we discuss how exogenously transplanted cells also secrete paracrine factors, which may be more advantageous in mediating cardiac repair by regulating endogenous factors that play direct and important roles in the regulation of neovascularization, fibrosis, inflammation, cardiac myocyte survival, contractility and bioenergetics and endogenous repair.

### Cell-derived paracrine factors and neovascularization

Review of the literature indicates, regardless of whether 'stem' or 'progenitor' cells consist of a mixture of several cell populations or selected subpopulations, that these cells have the capacity to mediate neovascularization. Kamihata *et al*. [26] have shown that bone marrow mononuclear cells (BM-MNCs), which consist of numerous different types of stem cells, transplanted into ischemic myocardium mediate angiogenesis via increased expression of angiogenic ligands and cytokines such as basic fibroblast growth factor (bFGF), vascular endothelial growth factor (VEGF), angiopoietin-1 (Ang-1), interleukin-1 beta (IL-1β), and tumor necrosis factor-alpha (TNF-α). Bone marrow derived stem cells (BMSCs) expressing c-kit and Sca-1 subjected to preconditioning (anoxic conditions) expressed increased amounts of activated Akt and activated eNOS, and secreted higher levels of VEGF, bFGF, insulin growth factor (IGF), and stromal cell derived factor-1 (SDF-1) compared to cells cultured under normal culture conditions, and the myocardial transplantation of these preconditioned cells led to increased blood vessel density [27]. Using MSCs, Kinnaird *et al*. [28] demonstrated the release of several angiogenic factors, such as VEGF, bFGF, placental growth factor (PIGF), and monocyte chemoattractant protein-1 (MCP-1), into the culture media, and the injection of these cells led to an increase in vessel number without MSC incorporation in mature vessels. Using gene expression profiling, additional studies from this laboratory demonstrate that MSCs express bFGF, FGF-7, IL-1, IL-6, PIGF, transforming growth factor-beta (TGF-β), TNF-α, and VEGF, which was augmented in response to hypoxia. This increased gene expression paralleled increased secreted protein levels of VEGF, bFGF, IL-6, PIGF, MCP-1, platelet-derived growth factor (PDGF), Ang-1, plasminogen activator (PA), and metalloproteinase-9 (MMP-9) [29]. In the heart, the intramyocardial injection of MSCs led to the *in vivo *upregulation of bFGF, VEGF, and SDF-1α, and led to increased vessel density after myocardial infarction (MI) [30]. Overexpression of VEGF in MSCs also led to increased capillary density following MI [31], suggesting that stem cells may be modulated to overexpress a variety of key factors that may further enhance their capacity to promote neovascularization in the heart.

Another population of bone marrow-derived stem cells, MAPCs, was also found to secrete factors such as VEGF, MCP-1, PDGF-BB, and TGF-β, and the authors postulated that this increase in angiogenic factors led to increased vascularity after the intramyocardial injection following MI [32]. Specifically, PDGF-BB and TGF-β may act in an autocrine manner on MAPCs to promote their differentiation into a smooth muscle cell-like phenotype [33]. Moreover, a novel clonally expandable population of BMSCs that did not express markers defining MSCs, HSCs, or MAPCs was also capable of secreting paracrine factors such as VEGF, hepatocyte growth factor (HGF), bFGF, PDGF-B, SDF-1α, Ang-1, Ang-2, and IGF-1, leading to therapeutic neovascularization [34].

Other cell types with angiogenic potential are those isolated and cultured from the circulating pool of mononuclear cells, and these cells express factors that are pro-angiogenic [35]. Specifically, we demonstrated significantly higher mRNA levels of VEGF-A, VEGF-B, SDF-1, and IGF-1 in cultured myeloid EPCs compared to adult endothelial cells, which paralleled significant release of VEGF, SDF-1, and IGF-1 protein into the cell culture supernatant. The paracrine effect of these cells could also be detected *in vivo *such that, in ischemic limbs, VEGF is also released from recruited human EPCs. As a functional consequence, conditioned medium of EPCs induced a strong migratory response of mature endothelial cells, which was significantly inhibited by VEGF and SDF-1 neutralizing antibodies. Taken together, EPCs exhibit a high expression of angiogenic growth factors that have a direct effect on mature endothelial cell migration and lead to improved neovascularization after ischemia.

Altogether, these studies suggest that several types of bone marrow-derived cells, consisting of either a mixture of different types of cells, or selected subpopulations of stem cells and circulating progenitor cells, have the capacity to express and secrete paracrine factors that lead to increased neovascularization following ischemia. Although these studies demonstrate an increased release of paracrine factors into the culture media and increased tissue expression of these paracrine factors upon cell transplantation, only some of the studies address whether these paracrine factors are released from the *in vivo *transplanted cells or whether the transplanted cells modulate endogenous tissue cytokine and growth factor levels. In a recent study, Cho *et al*. [36], demonstrated that the intramyocardial injection of human EPCs into mice led to the upregulation of a variety of angiogenic and anti-apoptotic factors, such as VEGF-A, FGF-2, IGF-1, HGF, Ang-1, and SDF-1, and these cells led to sustained upregulation of host endogenous factors, such as VEGF-A, FGF-2, Ang-1, Ang-2, PIGF, HGF, IGF-1, PDGF-B, and SDF-1, strongly suggesting that these endogenous factors may have contributed to the EPC-induced cardiac protection. Furthermore, Tateno *et al*. [37], using a hind limb ischemia model, demonstrated using IL-1β-deficient mice that the inability of muscle cells to secrete IL-1β reduced induction of angiogenic factors and impaired the neovascularization induced by BM-MNC transplantation. Regardless of whether the effects on elevated perfusion or neovascularization of ischemic tissue are due to paracrine factors from the stem cells themselves or whether these paracrine factors modulate endogenous cells to release factors promoting neovascularization, it is clear that transplanted BMSCs have a vasculogenic capacity and have the ability to improve function. However, it is becoming increasingly clear that the effects of stem cell-derived paracrine factors is not limited to their vasculogenic capacity, but also to their ability to modulate other mechanisms known to be involved in the development of heart failure.

### Cell-derived paracrine factors and cardiac myocyte protection

The protection of the cardiac myocyte from cell death has remained an attractive target for many therapeutic treatments for heart disease. Apoptosis, also known as programmed cell death, and necrosis play major roles in mediating ischemic injury and tissue remodeling. Thus, the possibility that cell therapy leads to protection against death of the cardiac muscle brings a new mechanism of the beneficial effects of stem cell therapy into focus. The measurement of paracrine factors from cultured stem cells as well as enhancement strategies provide evidence that stem cell-derived factors act directly to protect against cardiac myocyte cell death. Specifically, VEGF, bFGF, IGF, and SDF-1 were shown to be secreted by anoxic BMSCs, and the cell supernatants or the transplantation of these cells led to a decrease in cardiac myocyte apoptosis *in vitro *and *in vivo *and led to an upregulation of the well-known anti-apoptotic protein Bcl-2 in cardiac myocytes [27,38]. Furthermore, the overexpression of some of these factors in the heart has further substantiated their protective role in the heart after injury. For example, the intramyocardial injection of adenoviruses overexpressing VEGF led to decreased infarct size and increased expression of Bcl-2 [39], and injection of human recombinant bFGF also prevented ischemia-induced myocardial death and increased expression of Bcl-2 [40]. In addition, enhancement strategies targeted at improving the *in vivo *survival of the stem cells themselves has also proven to decrease cardiac myocyte death via the secretion of paracrine factors. The overexpression of the survival protein Akt in MSCs led to a decrease in cardiac myocyte apoptosis *in vitro *and their myocardial transplantation led to a decrease in infarct size [41-44]. In addition to secreting VEGF, bFGF, IGF, and SDF-1, Akt-overexpressing MSCs also secrete HGF, thymosin β4 (TB4), and secreted frizzled related protein 2 (Sfrp2) [45]. HGF has been shown to be protective in acute MI [46], and specifically anti-apoptotic, as shown by HGF gene transfer [47], intravenous HGF treatment [48], or its overexpression in transplanted MSCs [44]. TB4, a G-actin sequestering peptide, was also shown to directly promote survival of embryonic and postnatal cardiac myocytes in culture, and after coronary artery ligation in mice, TB4 treatment resulted in enhanced myocardial survival [49]. Sfrp2 released from Akt-overexpressing MSCs also leads to a decrease in cell death of isolated hypoxic cardiac myocytes in culture and specifically blocks the pro-apoptotic effects of Wnt3a [45].

Altogether, these studies demonstrate that paracrine factors from exogenously transplanted BMSCs expressing c-kit and Sca-1, or transplanted MSCs aid in prevention of cardiac myocyte cell death and, thus, in the preservation of muscle mass. However, the exact molecular pathways leading to this protection are not well defined and are likely to involve modulation of both caspase-dependent and caspase-independent pathways of cell death. This additionally discovered benefit of stem cell therapy for the treatment of the heart following injury may not be limited to cardiac myocyte protection. Since myocardial injury leads to an infiltration of inflammatory cells and upregulation of cytokines, stem cell therapy may additionally target these pathways involved in pathophysiological remodeling.

### Cell-derived paracrine factors and inflammation

There are two forms of immunity, innate and adaptive. Innate immune responses are phylogenetically conserved and initiate a quick response against a pathogen or myocardial injury, whereas adaptive immune responses involve antigen recognition and subsequent antibody generation. The regulation of these two types of immune responses in the heart involves upregulation and interaction of pro-inflammatory and anti-inflammatory cytokines. In response to stress, the heart increases expression of a variety of pro-inflammatory and anti-inflammatory cytokines, which play a dual role in the heart. Initial inflammatory cytokine expression is necessary for maintaining homeostatic responses within the heart after stress or injury; however, the dysregulation and sustained upregulation of certain cytokines ultimately leads to adverse remodelling and heart failure [50]. The specific regulation of expression and concentration of both pro-inflammatory and anti-inflammatory cytokines and their specific interactions is complex and incompletely understood in the heart, but may comprise a mechanism underlying the beneficial effects of stem cell therapy. There is increasing evidence that stem cells themselves, specifically MSCs, secrete a variety of pro-inflammatory and anti-inflammatory cytokines and that these cytokines may directly act to limit deleterious, sustained endogenous inflammation in the heart. Thus, administration of MSCs led to a downregulation of the cytokines TNF-α, IL-1β and IL-6, which are known to be involved in adverse LV remodelling [51]. Furthermore, the transplantation of MSCs attenuated myocardial dysfunction in a rat model of acute myocarditis [52]. Specifically, the authors demonstrated that MSC transplantation led to a decrease in CD68-positive inflammatory cells and decreased MCP-1 expression. Furthermore, the MSC-derived conditioned media protected isolated adult rat cardiac myocytes from MCP-1-induced injury, suggesting that the anti-inflammatory effects were due to paracrine factors released from these stem cells. Clearly, MSCs seem to possess anti-inflammatory properties, specifically through cytokine expression and infiltration of inflammatory cells, but these effects may not influence all aspects of innate immunity. The infiltration of neutrophils after MI, as measured by myeloid peroxidise (MPO) activity, did not appear to differ between intramyocardially transplanted singly cloned MSCs, unselected MSCs, BM-MNCs, or peripheral blood mononuclear cells (PBMNCs) [53]. While most studies focused on defining the role of adaptive immunity in the heart use models of autoimmune myocarditis or organ transplantation, it is becoming increasingly clear that T lymphocytes play a role in MI. Specifically, Varda-Bloom *et al*. [54] have shown that infiltration of T lymphocytes into the heart following MI, and *in vitro *co-culture of T lymphocytes from post-MI rats with isolated cardiac myocytes from a non-infarcted rat heart resulted in cytotoxicity of the cardiac myocytes. Accordingly, it is well established that MSCs play a role in suppressing adaptive immune responses. Specifically, MSCs suppressed T lymphocyte proliferation and MSCs co-cultured with purified subpopulations of immune cells altered cytokine secretion and induced a more anti-inflammatory phenotype [55,56]. It is clear that MSCs have a direct immunomodulatory role in the adaptive immune response, but the entire story of whether this immunomodulation occurs in the heart after MSC transplantation is far from complete; also, whether other stem cell populations play an immunomodulatory role in the heart has not been tested. These cells may directly modulate T lymphocyte function in the heart, leading to either protection against their cytotoxicity or, alternatively, modulation of their role in cardiac remodelling. Specifically, T lymphocytes co-cultured with cardiac fibroblasts led to an increase in cardiac fibroblast pro-collagen expression [57], suggesting that the *in vivo *suppression of T lymphocyte accumulation or function may also inhibit myocardial fibrosis. Thus, alterations in the innate and adaptive immune responses in the heart by transplanted stem cells may serve as another mechanism contributing to the improvement in LV function and the attenuation of adverse LV remodelling.

### Cell-derived paracrine factors and fibrosis

Most of the studies using stem cell therapy after MI have shown a reduction in fibrosis. However, in most studies it was not clearly defined whether the decreased fibrosis was simply due to replacement of dead myocardium or whether the paracrine factors released from these different subpopulations of stem cells may have had direct effects on the extracellular matrix. Nevertheless, in one study, a direct effect on fibrosis by stem cells was demonstrated; MSC-conditioned medium significantly attenuated proliferation of cardiac fibroblasts and upregulated elastin, myocardin and DNA-damage inducible transcript 3 [58]. Furthermore, MSC-conditioned medium significantly downregulated type I and III collagen expression, and significantly suppressed type III collagen promoter activity [58]. In a subsequent study, the intravenous injection of human multipotent stromal cells led to decreased fibrosis, and gene expression analysis of cultured cells revealed an upregulation of several matrix-modulating factors, such as matrix metalloproteinase-2 (MMP-2), tissue inhibitors of matrix metalloproteinases (TIMP)-1 and TIMP-2, and the matricellular proteins thrombospondin-1 and tenacin C, suggesting that these cells may have a direct effect on extracellular matrix remodeling [59]. Importantly, transplantation of MSCs led to decreased fibrosis in a rat model of dilated cardiomyopathy [60]. Specifically, the transplantation of these cells decreased MMP-2 and MMP-9 protein expression. Several paracrine factors released from stem cells, such as HGF, adrenomedullin (AM), TB4, and IL-1β, have previously been shown to directly reduce cardiac fibrosis. For example, adenovirus-mediated HGF gene transfer before ischemia led to decreased fibrosis [47], and treatment of dilated cardiomyopathic Syrian hamsters with HGF for three weeks suppressed cardiac fibrosis and led to a decrease in TGF-β1 and type I collagen expression [61]. AM administration also inhibited LV remodeling in heart failure [62] and specifically inhibited the proliferation of cardiac fibroblasts through a cAMP-dependent pathway [63]. TB4, harboring collagenase activity, has been shown to be important in cardiac healing after MI, and these effects may be mediated by its derivative, N-acetyl-seryl-aspartyl-lysyl-proline (Ac-SDKP). Ac-SDKP was reported to reduce LV fibrosis in hypertensive rats, reverse fibrosis and have collagenase activity, similar to TB4 [64]. IL-1β, also secreted from several populations of stem cells, has a direct anti-proliferative effect on cardiac fibroblasts [65]. Not only do the paracrine factors released from MSCs modulate myocardial fibrosis, but also cytokine or growth factor preconditioning of these MSCs leads suppression of fibrosis. For example, myocardially transplanted MSCs preconditioned with SDF-1 led to a decrease in fibrosis after 4 weeks MI which was abrogated using a CXCR4 antagonist [66]Moreover, the intramyocardial injection of TGF-β-pretreated CD117^+ ^cells led to a decrease in collagen fiber accumulation in the infarcted region [67]. Altogether, these studies suggest that stem cell-derived paracrine factors play a role in extracellular matrix remodeling that may contribute to the observed improvements in LV function after stem cell transplantation.

### Cell-derived paracrine factors and contractility and bioenergetics

Although few studies have investigated a direct role of bone marrow-derived cells on cardiac myocyte contractility and bioenergetic function, it is becoming increasingly clear that paracrine factors may act via this mechanism. In one study, the intravenous injection of MSCs preserved LV systolic function after MI without decreasing infarct size, suggesting that, in this case, MSCs did not protect against cardiac myocyte death but may have had a more direct effect on cardiac myocyte contractility [68]. Furthermore, incubation of supernatants from BM-MNCs with isolated adult cardiac myocytes cultured for 72 hours led to an increase in fractional shortening, the maximal rate of re-lengthening, the maximal rate of shortening, and the amplitude of the calcium ratio of the individual myocytes, suggesting that paracrine factors released from stem cells have a direct effect on the preservation of cardiac myocyte contractile capacity. This is further substantiated by an investigation determining whether myocardial stem cell transplantation leads to changes in enzymes that modulate intracellular calcium and mediate cardiac myocyte contraction. Using a population of heart-derived stem cells expressing Sca-1 but lacking CD31, it was shown that the intramyocardial injection of these cells following MI led to a higher phosphocreatine/ATP ratio compared to non-stem cell-treated hearts and higher creatine kinase-mt, creatine kinase-m, and mitochondrial ATPase-β, suggesting improved bioenergetic characteristics [69]. Furthermore, the intramyocardial injection of a stock of swine multipotent adult progenitor cells ('pMultistem cells') led to a recovery of border zone subendocardial phosphocreatine/ATP ratios and increased expression of creatine kinase-mt and creatine kinase-m isoforms after MI [70]. Altogether, these studies suggest that bone marrow-derived cells have the capacity to modulate calcium handling, and preserve cardiac contractility and bioenergetics. Whether these improvements directly limit structural and contractile abnormalities in heart disease or whether these improvements occur in parallel with, or in addition to, other stem cell-mediated beneficial effects remains to be determined.

### Cell-derived paracrine factors and endogenous repair

Another mechanism of cell therapy includes the ability of transplanted cells to promote endogenous repair, which may include modulation of cardiac-resident stem cells and epicardial progenitor cells by specifically regulating endogenous or stem cell-derived paracrine factors. One study demonstrated that cardiac stem cells and early committed cells expressing c-kit, MDR1, and Sca-1 express c-Met and IGF-1 receptors and synthesize and secrete corresponding ligands, HGF and IGF-1, suggesting that these factors may act in an autocrine manner to regulate the functionality of these cells [71]. Furthermore, since these cells express receptors for HGF and IGF-1, it is possible that paracrine factors secreted from transplanted BMSCs into the myocardium or transplanted circulating blood-derived progenitor cells may regulate the functionality of these cardiac resident stem cells, thereby leading to cardiac repair and protection. Consistent with this hypothesis, supernatants of myeloid EPCs were shown to induce migration of cardiac stem cells [72]. These preliminary data suggest that a complex cross-talk may exist between transplanted stem cells and the endogenous cardiac cell pool. The delineation of these pathways will be important for the future of stem cell therapy in heart failure.

### Paracrine factors from non-bone marrow derived stem cells

Stem cells from sources other than those derived from bone marrow are also the subject of numerous investigations. For example, mesenchymal stem cells can also be isolated from adipose tissue [73], muscle [74], umbilical cord blood [75], and a variety of other tissues [76-80]. It has also been suggested that MSCs can be found in all post-mitotic organs and tissues, including vessel walls [81]. It is not entirely clear whether these cells function identically in improving cardiac performance to MSCs derived from the bone marrow. Using a hind limb ischemia model, one study suggested that MSCs isolated from adipose tissue have a greater angiogenic capacity than MSCs isolated from bone marrow [82], and these cells had higher expression levels of MMP-3 and MMP-9. Alternatively, these cells, derived from either adipose tissue or bone marrow, have similar capacities to secrete paracrine factors. Cultured adipose derived stem cells (ASCs) secrete large amounts of VEGF, HGF, and TGF-β, and high levels of VEGF when subjected to hypoxic conditions. The conditioned media from hypoxic ASCs significantly increased endothelial cell growth and reduced endothelial cell apoptosis, and transplantation of these cells into ischemic hind limbs led to improved perfusion, suggesting paracrine factors from these cells promote neovascularization [83]. Interestingly, the ability of ASCs to promote survival, proliferation, and migration of mature and progenitor endothelial cells *in vitro *and to promote reperfusion in a mouse hind limb ischemia model appears to be dependent on HGF [84]. In the heart, the transplantation of adult progenitor cells derived from either adipose tissue or bone marrow led to a decrease in myocardial pro-inflammatory and pro-apoptotic signaling and, thus, to cardiac protection against ischemia [85]. As researchers continue to study and compare stem cells from diverse organs and tissues, it will become evident whether overlapping or divergent mechanisms exist between these cells, and which paracrine factors are released from cells. These ongoing studies will prove beneficial in identifying which cell type may be optimal for the treatment of ischemic diseases and, ultimately, for the treatment of heart failure.

## Conclusion

**Figure 1:**
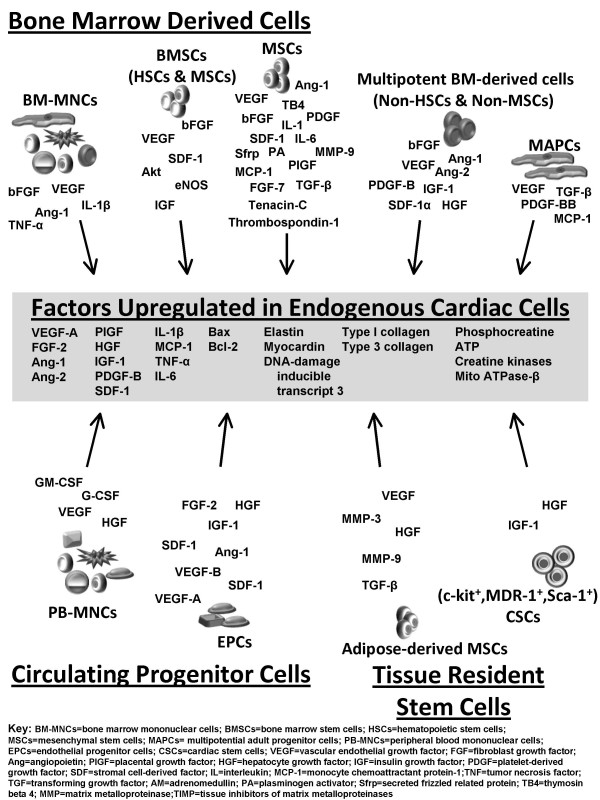
Actions of stem cell derived paracrine factors on the heart. Stem cells transplanted into the myocardium release numerous factors that may act in an autocrine manner or paracrine manner to modulate the implanted cells themselves, or the endogenous cells of the heart, including cardiac myocytes, fibroblasts, endothelial cells, vascular smooth muscle cells, and cardiac stem cells. These factors include a variety of growth factors, cytokines, and extracellular matrix proteins that may lead to upregulation of several endogenous growth factors, cytokines, and extracellular matrix proteins, thereby tightly regulating neovascularization, protection against cell death, inflammation, fibrosis, contractility, bioenergetics, and endogenous repair. Regulation of these processes, either singly or in combination, by stem cell transplantation ultimately leads to improvement in left ventricular function following myocardial infarction. Future research in discovering novel stem-derived paracrine factors and their precise mechanistic roles in heart repair and fibrosis may ultimately lead to the generation of novel therapeutic agents for the treatment of heart failure.

Paracrine factors from stem cells transplanted into the myocardium play an important role in modulating LV remodeling after an ischemic injury. It should be noted that the evidence supporting this idea is based on numerous studies in animal models. The contribution of the paracrine effects in clinical trials (compared to cell intrinsic functions) cannot be exactly determined as long as autologous cells are implanted and the availability of tissue restricts information on local cytokine production. However, the clinically used cell populations, namely total bone marrow nuclear cells [26,86], cultured endothelial progenitor cells [36,72], and MSCs [28,29,41-45], were all shown to release paracrine factors when transplanted into ischemia models. In the future, not only will it be important to determine which paracrine factors are up- or down-regulated, but also to characterize the spatio-temporal release and the local concentrations produced by the injected or infused cell populations. Furthermore, an understanding of synergistic or additive interactions between these factors is crucial, as well as of whether these factors act on one or a combination of mechanisms that lead to heart failure. This may ultimately lead to the generation of pharmacological agents that can be used to treat heart failure, possibly negating the need for cell-based therapy altogether, (see Figure 1).

## Competing interests

The authors declare that they have no competing interests.

## Authors' contributions

JB drafted the review. SD conceived, designed, drafted, and edited the review.
